# 2-[3-(2-Chloro­phen­yl)-5-oxo-1,5-diphenyl­pentyl­idene]malononitrile

**DOI:** 10.1107/S1600536810050622

**Published:** 2010-12-11

**Authors:** Bai-Xiang Du, Jie Zhou, Yu-Ling Li, Xiang-Shan Wang

**Affiliations:** a School of Chemistry and Chemical Engineering, Xuzhou Normal University, Xuzhou Jiangsu 221116, People’s Republic of China

## Abstract

In the title compound, C_26_H_19_ClN_2_O, the 2-chloro­phenyl group forms dihedral angles of 59.6 (1) and 31.9 (1)° with the phenyl rings. The two phenyl rings are inclined at a dihedral angle of 32.9 (1)° with respect to each other. In the crystal, an inter­molecular C—H⋯N hydrogen bond links the mol­ecules into a polymeric chain running along the *c* axis.

## Related literature

For water as an attractive medium for organic reactions, see: Breslow (1991 ▶). For a related structure, see: Zhou *et al.* (2007 ▶).
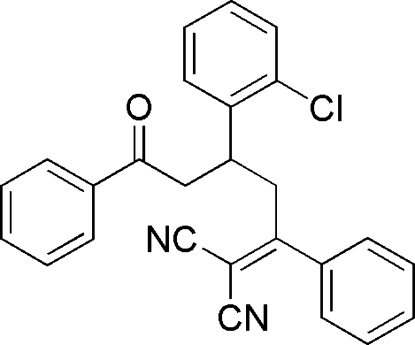


## Experimental

### 

#### Crystal data

C_26_H_19_ClN_2_O
*M* *_r_* = 410.88
Orthorhombic, 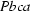

*a* = 12.4450 (3) Å
*b* = 14.3866 (3) Å
*c* = 24.1913 (5) Å
*V* = 4331.24 (16) Å^3^
*Z* = 8
Mo *K*α radiation
μ = 0.20 mm^−1^
*T* = 296 K
0.50 × 0.39 × 0.29 mm

#### Data collection

Bruker SMART CCD area-detector diffractometer
34193 measured reflections
5019 independent reflections
2972 reflections with *I* > 2σ(*I*)
*R*_int_ = 0.042

#### Refinement

*R*[*F* ^2^ > 2σ(*F* ^2^)] = 0.049
*wR*(*F* ^2^) = 0.153
*S* = 1.04
5019 reflections
271 parameters
H-atom parameters constrained
Δρ_max_ = 0.22 e Å^−3^
Δρ_min_ = −0.27 e Å^−3^

### 

Data collection: *SMART* (Bruker, 2001 ▶); cell refinement: *SAINT* (Bruker, 2001 ▶); data reduction: *SAINT*; program(s) used to solve structure: *SHELXS97* (Sheldrick, 2008 ▶); program(s) used to refine structure: *SHELXL97* (Sheldrick, 2008 ▶); molecular graphics: *SHELXTL* (Sheldrick, 2008 ▶); software used to prepare material for publication: *SHELXTL*.

## Supplementary Material

Crystal structure: contains datablocks global, I. DOI: 10.1107/S1600536810050622/pv2363sup1.cif

Structure factors: contains datablocks I. DOI: 10.1107/S1600536810050622/pv2363Isup2.hkl

Additional supplementary materials:  crystallographic information; 3D view; checkCIF report

## Figures and Tables

**Table 1:** Hydrogen-bond geometry (Å, °)

*D*—H⋯*A*	*D*—H	H⋯*A*	*D*⋯*A*	*D*—H⋯*A*
C8—H8*A*⋯N1^i^	0.97	2.62	3.502 (3)	152

Symmetry code: (i) 
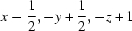
.

**Fig. 1.**
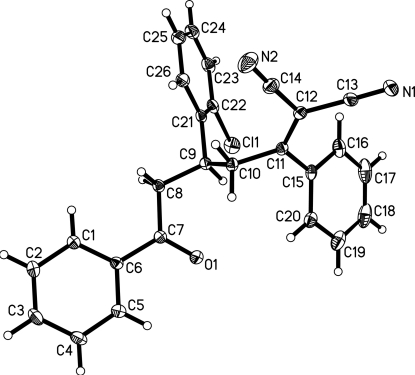
The molecular structure drawing for the title compound; displacement ellipsoids are drawn at 20% probability level.

**Fig. 2.**
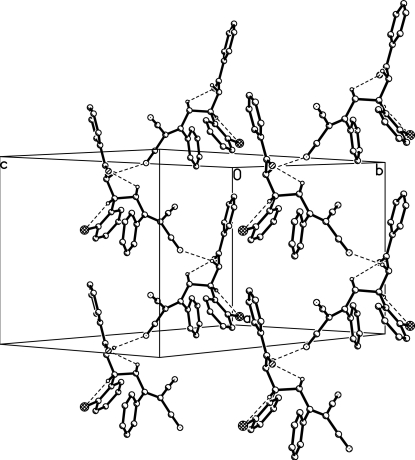
The molecular packing diagram of the title compound showing intermolecular and intramolecular interactions by dashed lines.

## supplementary crystallographic information

### Comment

There has been a growing recognition that water has become an attractive medium for many organic reactions (Breslow, 1991). Recently, we have demonstrated the Michal addition reaction between 2-(1-phenylethylidene)malononitrile and 1-phenyl-3-(2-chlorophenyl)propen-1-one in water without a catalyst and synthesized the title compound which is reported in this article.

In the title compound (Fig. 1), 2-chlorophenyl group forms dihedral angles of 59.6 (1) and 31.9 (1) ° with benzene rings (C1—C6) and (C15—C20), respectively. The two benzene rings are inclined with respect to each other at a dihedral angle of 32.9 (1) °. There is an intermolecular hydrogen bond C8—H8A···N1 (Table 1) resulting in a polymeric chain along the *c*-axis. In addition, two intramolecular interactions further stabilize the structure (Fig. 2).

### Experimental

The title compound was prepared by the reaction of 2-(1-phenylethylidene)malononitrile (0.168 g, 1.0 mmol) and 1-phenyl-3-(2-chlorophenyl)propen-1-one (0.242 g, 1.0 mmol) in water (10 ml) at reflux for 14 h (yield 82%, mp. 438–439 K). Crystals of the title compound suitable for X-ray diffraction were obtained by slow evaporation of a dimethylformamide solution.

### Refinement

The H atoms were calculated geometrically and refined as riding, with C—H = 0.93–0.98 Å, and with *U*_iso_(H) = 1.2*U*_eq_(parent atom).

### Crystal data

**Table:**

C_26_H_19_ClN_2_O	*D*_x_ = 1.260 Mg m^−^^3^
*M**_r_* = 410.88	Melting point = 438–439 K
Orthorhombic, *P**b**c**a*	Mo *K*α radiation, λ = 0.71073 Å
Hall symbol: -P 2ac 2ab	Cell parameters from 5457 reflections
*a* = 12.4450 (3) Å	θ = 2.7–22.0°
*b* = 14.3866 (3) Å	µ = 0.20 mm^−^^1^
*c* = 24.1913 (5) Å	*T* = 296 K
*V* = 4331.24 (16) Å^3^	Block, orange
*Z* = 8	0.50 × 0.39 × 0.29 mm
*F*(000) = 1712	

### Data collection

**Table:**

Bruker SMART CCD area-detector diffractometer	2972 reflections with *I* > 2σ(*I*)
Radiation source: fine-focus sealed tube	*R*_int_ = 0.042
graphite	θ_max_ = 27.6°, θ_min_ = 1.7°
φ and ω scans	*h* = −14→16
34193 measured reflections	*k* = −16→18
5019 independent reflections	*l* = −31→31

### Refinement

**Table:**

Refinement on *F*^2^	Primary atom site location: structure-invariant direct methods
Least-squares matrix: full	Secondary atom site location: difference Fourier map
*R*[*F*^2^ > 2σ(*F*^2^)] = 0.049	Hydrogen site location: inferred from neighbouring sites
*wR*(*F*^2^) = 0.153	H-atom parameters constrained
*S* = 1.04	*w* = 1/[σ^2^(*F*_o_^2^) + (0.0713*P*)^2^ + 0.4979*P*] where *P* = (*F*_o_^2^ + 2*F*_c_^2^)/3
5019 reflections	(Δ/σ)_max_ = 0.001
271 parameters	Δρ_max_ = 0.22 e Å^−^^3^
0 restraints	Δρ_min_ = −0.27 e Å^−^^3^

### Special details

**Table:**

Geometry. All e.s.d.'s (except the e.s.d. in the dihedral angle between two l.s. planes) are estimated using the full covariance matrix. The cell e.s.d.'s are taken into account individually in the estimation of e.s.d.'s in distances, angles and torsion angles; correlations between e.s.d.'s in cell parameters are only used when they are defined by crystal symmetry. An approximate (isotropic) treatment of cell e.s.d.'s is used for estimating e.s.d.'s involving l.s. planes.
Refinement. Refinement of *F*^2^ against ALL reflections. The weighted *R*-factor *wR* and goodness of fit *S* are based on *F*^2^, conventional *R*-factors *R* are based on *F*, with *F* set to zero for negative *F*^2^. The threshold expression of *F*^2^ > σ(*F*^2^) is used only for calculating *R*-factors(gt) *etc*. and is not relevant to the choice of reflections for refinement. *R*-factors based on *F*^2^ are statistically about twice as large as those based on *F*, and *R*- factors based on ALL data will be even larger.

### Fractional atomic coordinates and isotropic or equivalent isotropic displacement parameters (Å^2^)

**Table:**

	*x*	*y*	*z*	*U*_iso_*/*U*_eq_	
Cl1	0.39311 (5)	0.00962 (5)	0.68110 (3)	0.0971 (3)	
C9	0.21419 (13)	0.09876 (11)	0.61061 (7)	0.0520 (4)	
H9A	0.2428	0.1139	0.6473	0.062*	
C8	0.10322 (13)	0.05493 (12)	0.61832 (8)	0.0560 (4)	
H8A	0.0692	0.0488	0.5824	0.067*	
H8B	0.1121	−0.0071	0.6334	0.067*	
C21	0.29056 (14)	0.03111 (11)	0.58342 (8)	0.0556 (5)	
C10	0.20485 (14)	0.18975 (11)	0.57755 (8)	0.0557 (4)	
H10A	0.1812	0.1751	0.5403	0.067*	
H10B	0.1501	0.2284	0.5945	0.067*	
C11	0.30676 (14)	0.24393 (10)	0.57417 (8)	0.0544 (4)	
C6	−0.08495 (15)	0.08221 (13)	0.66065 (8)	0.0572 (5)	
C22	0.37359 (15)	−0.01196 (12)	0.61140 (10)	0.0678 (5)	
C12	0.34607 (16)	0.27063 (12)	0.52452 (8)	0.0625 (5)	
C15	0.35978 (16)	0.27215 (12)	0.62569 (8)	0.0610 (5)	
O1	0.06454 (13)	0.17514 (13)	0.68188 (7)	0.1005 (6)	
C7	0.03017 (16)	0.10937 (13)	0.65567 (8)	0.0615 (5)	
N1	0.50586 (17)	0.38254 (14)	0.51232 (9)	0.0950 (6)	
C13	0.43597 (18)	0.33273 (14)	0.51864 (9)	0.0727 (6)	
C24	0.4281 (2)	−0.09442 (16)	0.53127 (15)	0.0962 (8)	
H24A	0.4745	−0.1357	0.5137	0.115*	
C1	−0.12962 (17)	0.00934 (14)	0.63200 (10)	0.0752 (6)	
H1A	−0.0876	−0.0250	0.6076	0.090*	
C20	0.2988 (2)	0.30836 (13)	0.66865 (8)	0.0747 (6)	
H20A	0.2244	0.3114	0.6654	0.090*	
C16	0.4708 (2)	0.26488 (16)	0.63205 (11)	0.0878 (7)	
H16A	0.5128	0.2394	0.6041	0.105*	
C26	0.27768 (18)	0.00750 (12)	0.52708 (10)	0.0721 (6)	
H26A	0.2221	0.0341	0.5069	0.087*	
C14	0.2967 (2)	0.24452 (15)	0.47332 (11)	0.0852 (7)	
C5	−0.14936 (19)	0.13109 (16)	0.69652 (9)	0.0805 (6)	
H5A	−0.1202	0.1799	0.7167	0.097*	
C23	0.44248 (17)	−0.07472 (15)	0.58579 (13)	0.0880 (7)	
H23A	0.4976	−0.1028	0.6057	0.106*	
C25	0.3458 (2)	−0.05403 (15)	0.50173 (11)	0.0889 (7)	
H25A	0.3365	−0.0685	0.4646	0.107*	
C2	−0.2366 (2)	−0.01304 (19)	0.63923 (12)	0.0981 (8)	
H2A	−0.2663	−0.0624	0.6197	0.118*	
C3	−0.2987 (2)	0.0365 (2)	0.67466 (11)	0.0947 (8)	
H3A	−0.3705	0.0207	0.6795	0.114*	
C4	−0.25626 (19)	0.1091 (2)	0.70305 (10)	0.0921 (7)	
H4A	−0.2992	0.1438	0.7268	0.111*	
C18	0.4571 (4)	0.3335 (2)	0.72144 (15)	0.1327 (15)	
H18A	0.4900	0.3550	0.7535	0.159*	
N2	0.2593 (3)	0.22584 (17)	0.43153 (10)	0.1295 (10)	
C17	0.5177 (3)	0.2960 (2)	0.68043 (17)	0.1288 (13)	
H17A	0.5917	0.2912	0.6849	0.155*	
C19	0.3479 (3)	0.33966 (17)	0.71595 (11)	0.1089 (10)	
H19A	0.3068	0.3651	0.7443	0.131*	

### Atomic displacement parameters (Å^2^)

**Table:**

	*U*^11^	*U*^22^	*U*^33^	*U*^12^	*U*^13^	*U*^23^
Cl1	0.0767 (4)	0.1071 (5)	0.1076 (5)	0.0193 (3)	−0.0259 (3)	0.0111 (4)
C9	0.0472 (10)	0.0470 (9)	0.0617 (10)	0.0028 (7)	−0.0016 (8)	0.0037 (7)
C8	0.0494 (10)	0.0497 (9)	0.0687 (11)	0.0002 (8)	0.0018 (8)	0.0032 (8)
C21	0.0473 (10)	0.0425 (8)	0.0771 (12)	−0.0043 (7)	0.0066 (9)	0.0054 (8)
C10	0.0528 (11)	0.0448 (9)	0.0695 (11)	0.0022 (7)	−0.0056 (9)	0.0027 (8)
C11	0.0537 (11)	0.0370 (8)	0.0724 (12)	0.0059 (7)	−0.0006 (9)	0.0003 (8)
C6	0.0515 (11)	0.0589 (10)	0.0611 (11)	0.0043 (8)	0.0024 (8)	0.0024 (8)
C22	0.0484 (11)	0.0523 (10)	0.1027 (16)	0.0010 (8)	0.0065 (10)	0.0106 (10)
C12	0.0688 (12)	0.0474 (9)	0.0714 (12)	0.0021 (9)	0.0110 (10)	−0.0048 (9)
C15	0.0651 (12)	0.0435 (9)	0.0744 (13)	−0.0092 (8)	−0.0103 (10)	0.0107 (8)
O1	0.0712 (10)	0.1136 (13)	0.1167 (13)	−0.0219 (9)	0.0164 (9)	−0.0566 (11)
C7	0.0538 (11)	0.0636 (11)	0.0671 (12)	−0.0002 (9)	0.0001 (9)	−0.0066 (9)
N1	0.0909 (15)	0.0822 (13)	0.1118 (16)	−0.0147 (11)	0.0316 (12)	0.0016 (11)
C13	0.0747 (14)	0.0588 (12)	0.0846 (15)	0.0030 (11)	0.0227 (12)	−0.0024 (10)
C24	0.0767 (17)	0.0579 (13)	0.154 (3)	0.0045 (12)	0.0416 (18)	−0.0098 (15)
C1	0.0578 (13)	0.0751 (13)	0.0927 (15)	−0.0047 (10)	0.0077 (11)	−0.0159 (11)
C20	0.0963 (17)	0.0591 (12)	0.0689 (13)	−0.0124 (11)	−0.0060 (12)	0.0020 (10)
C16	0.0694 (15)	0.0785 (14)	0.1154 (19)	−0.0124 (12)	−0.0217 (14)	0.0221 (13)
C26	0.0755 (14)	0.0498 (10)	0.0912 (15)	0.0016 (9)	0.0152 (12)	0.0004 (10)
C14	0.118 (2)	0.0643 (13)	0.0733 (15)	−0.0065 (12)	0.0138 (14)	−0.0044 (11)
C5	0.0680 (14)	0.0882 (15)	0.0854 (15)	−0.0005 (12)	0.0126 (12)	−0.0168 (12)
C23	0.0545 (13)	0.0653 (13)	0.144 (2)	0.0088 (10)	0.0151 (14)	0.0067 (15)
C25	0.1003 (19)	0.0621 (13)	0.1042 (18)	−0.0081 (13)	0.0309 (15)	−0.0117 (12)
C2	0.0648 (15)	0.1060 (19)	0.124 (2)	−0.0228 (13)	0.0044 (15)	−0.0243 (16)
C3	0.0535 (14)	0.124 (2)	0.1068 (19)	−0.0112 (14)	0.0122 (13)	0.0044 (17)
C4	0.0614 (14)	0.1157 (19)	0.0992 (17)	0.0068 (14)	0.0222 (13)	−0.0104 (15)
C18	0.194 (4)	0.107 (2)	0.098 (2)	−0.067 (3)	−0.067 (3)	0.0279 (19)
N2	0.206 (3)	0.1064 (17)	0.0766 (15)	−0.0285 (19)	−0.0054 (16)	−0.0090 (13)
C17	0.110 (3)	0.121 (3)	0.155 (3)	−0.048 (2)	−0.071 (2)	0.050 (2)
C19	0.173 (3)	0.0812 (16)	0.0730 (16)	−0.0366 (19)	−0.0189 (19)	0.0037 (12)

### Geometric parameters (Å, °)

**Table:**

Cl1—C22	1.732 (3)	C24—C25	1.378 (4)
C9—C21	1.511 (2)	C24—H24A	0.9300
C9—C8	1.530 (2)	C1—C2	1.381 (3)
C9—C10	1.538 (2)	C1—H1A	0.9300
C9—H9A	0.9800	C20—C19	1.373 (3)
C8—C7	1.502 (3)	C20—H20A	0.9300
C8—H8A	0.9700	C16—C17	1.382 (4)
C8—H8B	0.9700	C16—H16A	0.9300
C21—C22	1.382 (3)	C26—C25	1.371 (3)
C21—C26	1.414 (3)	C26—H26A	0.9300
C10—C11	1.491 (2)	C14—N2	1.145 (3)
C10—H10A	0.9700	C5—C4	1.377 (3)
C10—H10B	0.9700	C5—H5A	0.9300
C11—C12	1.353 (2)	C23—H23A	0.9300
C11—C15	1.468 (3)	C25—H25A	0.9300
C6—C1	1.374 (3)	C2—C3	1.357 (4)
C6—C5	1.375 (3)	C2—H2A	0.9300
C6—C7	1.490 (3)	C3—C4	1.356 (4)
C22—C23	1.391 (3)	C3—H3A	0.9300
C12—C14	1.433 (3)	C4—H4A	0.9300
C12—C13	1.439 (3)	C18—C17	1.357 (5)
C15—C20	1.388 (3)	C18—C19	1.369 (5)
C15—C16	1.394 (3)	C18—H18A	0.9300
O1—C7	1.217 (2)	C17—H17A	0.9300
N1—C13	1.137 (3)	C19—H19A	0.9300
C24—C23	1.361 (4)		
			
C21—C9—C8	110.83 (13)	C25—C24—H24A	119.6
C21—C9—C10	111.67 (14)	C6—C1—C2	120.3 (2)
C8—C9—C10	110.24 (13)	C6—C1—H1A	119.9
C21—C9—H9A	108.0	C2—C1—H1A	119.9
C8—C9—H9A	108.0	C19—C20—C15	120.2 (3)
C10—C9—H9A	108.0	C19—C20—H20A	119.9
C7—C8—C9	113.89 (15)	C15—C20—H20A	119.9
C7—C8—H8A	108.8	C17—C16—C15	119.2 (3)
C9—C8—H8A	108.8	C17—C16—H16A	120.4
C7—C8—H8B	108.8	C15—C16—H16A	120.4
C9—C8—H8B	108.8	C25—C26—C21	121.1 (2)
H8A—C8—H8B	107.7	C25—C26—H26A	119.5
C22—C21—C26	116.69 (18)	C21—C26—H26A	119.5
C22—C21—C9	123.08 (18)	N2—C14—C12	177.7 (3)
C26—C21—C9	120.21 (17)	C6—C5—C4	121.2 (2)
C11—C10—C9	114.15 (14)	C6—C5—H5A	119.4
C11—C10—H10A	108.7	C4—C5—H5A	119.4
C9—C10—H10A	108.7	C24—C23—C22	119.1 (2)
C11—C10—H10B	108.7	C24—C23—H23A	120.5
C9—C10—H10B	108.7	C22—C23—H23A	120.5
H10A—C10—H10B	107.6	C26—C25—C24	120.0 (3)
C12—C11—C15	120.85 (17)	C26—C25—H25A	120.0
C12—C11—C10	120.33 (17)	C24—C25—H25A	120.0
C15—C11—C10	118.72 (16)	C3—C2—C1	120.4 (2)
C1—C6—C5	118.21 (19)	C3—C2—H2A	119.8
C1—C6—C7	123.25 (17)	C1—C2—H2A	119.8
C5—C6—C7	118.53 (18)	C2—C3—C4	120.2 (2)
C21—C22—C23	122.3 (2)	C2—C3—H3A	119.9
C21—C22—Cl1	120.10 (16)	C4—C3—H3A	119.9
C23—C22—Cl1	117.62 (18)	C3—C4—C5	119.7 (2)
C11—C12—C14	122.55 (18)	C3—C4—H4A	120.2
C11—C12—C13	123.06 (19)	C5—C4—H4A	120.2
C14—C12—C13	114.25 (19)	C17—C18—C19	120.4 (3)
C20—C15—C16	119.2 (2)	C17—C18—H18A	119.8
C20—C15—C11	119.59 (18)	C19—C18—H18A	119.8
C16—C15—C11	121.2 (2)	C18—C17—C16	120.8 (3)
O1—C7—C6	119.99 (18)	C18—C17—H17A	119.6
O1—C7—C8	120.41 (18)	C16—C17—H17A	119.6
C6—C7—C8	119.60 (16)	C18—C19—C20	120.1 (3)
N1—C13—C12	177.8 (3)	C18—C19—H19A	120.0
C23—C24—C25	120.8 (2)	C20—C19—H19A	120.0
C23—C24—H24A	119.6		
			
C21—C9—C8—C7	167.52 (15)	C11—C12—C13—N1	−157 (6)
C10—C9—C8—C7	−68.3 (2)	C14—C12—C13—N1	19 (6)
C8—C9—C21—C22	−107.58 (19)	C5—C6—C1—C2	0.4 (3)
C10—C9—C21—C22	129.09 (17)	C7—C6—C1—C2	179.0 (2)
C8—C9—C21—C26	71.0 (2)	C16—C15—C20—C19	2.4 (3)
C10—C9—C21—C26	−52.3 (2)	C11—C15—C20—C19	−176.51 (18)
C21—C9—C10—C11	−63.88 (19)	C20—C15—C16—C17	−1.6 (3)
C8—C9—C10—C11	172.46 (15)	C11—C15—C16—C17	177.3 (2)
C9—C10—C11—C12	126.58 (17)	C22—C21—C26—C25	−1.0 (3)
C9—C10—C11—C15	−56.9 (2)	C9—C21—C26—C25	−179.72 (17)
C26—C21—C22—C23	0.7 (3)	C11—C12—C14—N2	166 (8)
C9—C21—C22—C23	179.38 (17)	C13—C12—C14—N2	−9(8)
C26—C21—C22—Cl1	−177.92 (13)	C1—C6—C5—C4	−1.1 (3)
C9—C21—C22—Cl1	0.7 (2)	C7—C6—C5—C4	−179.8 (2)
C15—C11—C12—C14	179.97 (18)	C25—C24—C23—C22	−0.9 (4)
C10—C11—C12—C14	−3.6 (3)	C21—C22—C23—C24	0.2 (3)
C15—C11—C12—C13	−4.6 (3)	Cl1—C22—C23—C24	178.89 (18)
C10—C11—C12—C13	171.84 (16)	C21—C26—C25—C24	0.4 (3)
C12—C11—C15—C20	132.33 (19)	C23—C24—C25—C26	0.6 (3)
C10—C11—C15—C20	−44.1 (2)	C6—C1—C2—C3	0.0 (4)
C12—C11—C15—C16	−46.5 (2)	C1—C2—C3—C4	0.5 (4)
C10—C11—C15—C16	137.02 (17)	C2—C3—C4—C5	−1.2 (4)
C1—C6—C7—O1	179.4 (2)	C6—C5—C4—C3	1.6 (4)
C5—C6—C7—O1	−2.0 (3)	C19—C18—C17—C16	1.1 (5)
C1—C6—C7—C8	−0.8 (3)	C15—C16—C17—C18	−0.1 (4)
C5—C6—C7—C8	177.80 (18)	C17—C18—C19—C20	−0.3 (4)
C9—C8—C7—O1	−8.8 (3)	C15—C20—C19—C18	−1.4 (4)
C9—C8—C7—C6	171.45 (16)		

### Hydrogen-bond geometry (Å, °)

**Table:**

*D*—H···*A*	*D*—H	H···*A*	*D*···*A*	*D*—H···*A*
C8—H8A···N1^i^	0.97	2.62	3.502 (3)	152.
C9—H9A···Cl1	0.98	2.53	3.083 (3)	115.
C10—H10B···O1	0.97	2.48	3.071 (4)	119.

Symmetry codes: (i) *x*−1/2, −*y*+1/2, −*z*+1.
